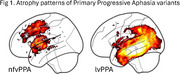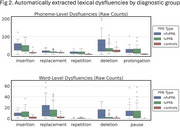# Automated Lexical Dysfluency Analysis to Differentiate Primary Progressive Aphasia Variants

**DOI:** 10.1002/alz70857_105719

**Published:** 2025-12-24

**Authors:** Jet MJ Vonk, Jiachen Lian, Zoe Ezzes, Lisa D. Wauters, Cheol Jun Cho, Brittany T Morin, Rian Bogley, Zachary Miller, Maria Luisa Mandelli, Gopala K Anumanchipalli, Maria Luisa Gorno Tempini, Diana Rodriguez

**Affiliations:** ^1^ University of California San Francisco (UCSF), San Francisco, CA, USA; ^2^ University of California Berkeley, Berkeley, CA, USA; ^3^ The University of Texas at Austin, Austin, TX, USA

## Abstract

**Background:**

The non‐fluent and logopenic variants of Primary Progressive Aphasia (nfvPPA, lvPPA;Figure 1) can be difficult to distinguish in early disease stages when expert clinicians need to correctly identify the specific nature of speech errors. NfvPPA is characterized by motor speech impairments and phonetic‐motoric errors, while lvPPA involves phonological errors like sound substitutions and transpositions. Automated speech recognition (ASR) systems are promising tools to aid clinicians in objectively analyzing speech and language in dementia. However, current tools are inadequate in identifying specific speech errors, particularly at the phoneme level, as they prioritize fluent transcription omitting dysfluencies. We hypothesized that our novel forced alignment‐based Scalable Speech Dysfluency Modeling Lightweight (SSDM‐L) overcomes these limitations by capturing phoneme‐ and word‐level disruptions, offering a data‐driven alternative to perceptual judgment.

**Method:**

We analyzed recordings of reading aloud the Grandfather passage from 31 individuals with nfvPPA, 67 lvPPA, and 26 controls. Ten dysfluency variables were extracted using SSDM‐L, including phoneme‐ and word‐level insertions, replacements, repetitions, deletions, phoneme prolongations, and pauses between words. Unlike conventional ASR, SSDM‐L incorporates a robust dysfluent‐ and time‐aware phoneme and word transcriber, phonetic‐aware subsequence aligner, and rule‐based dysfluency detector to accurately identify phonemic errors with high precision. Group differences were assessed via MANCOVA, adjusting for age, education, and disease severity (MMSE, CDR‐sum‐of‐boxes), and multiple comparisons. Backward stepwise logistic regression with repeated train‐test splits identified robust predictors, and 5‐fold cross‐validation evaluated model performance.

**Result:**

All features except word repetition distinguished controls from PPA groups (*p* < .001‐.012). NfvPPA and lvPPA differed in phoneme replacement (*p* = .036), phoneme deletions (*p* < .001), word replacement (*p* = .012), and word deletions (*p* < .001; Figure 2). These four features were also together selected as key predictors in logistic regression, yielding 81.6% accuracy (AUC = .761). Adding covariates yielded 80.5% accuracy (AUC = .843), while a covariates‐only model achieved 65.2% accuracy (AUC = .659).

**Conclusion:**

Differentiating nfvPPA and lvPPA relies on detailed phoneme‐level error characterization, traditionally requiring extensive expertise and manual effort. Our novel approach automates this process using a quick and easy‐to‐administer reading task, providing an objective, scalable, and clinically viable solution. By capturing dysfluencies missed by ASR, this method enhances diagnostic precision and reduces reliance on highly specialized clinicians, facilitating broader clinical adoption for differential diagnosis.